# ABI3BP is a prognosis biomarker related with clinicopathological features and immunity infiltration of lung tumor

**DOI:** 10.3389/fgene.2022.1085785

**Published:** 2023-01-19

**Authors:** Yan Feng, Xiaolei Han, Zhe Zhang, Han Qiao, Huaping Tang

**Affiliations:** ^1^ Department of Respiratory Medicine, Qingdao Municipal Hospital, Qingdao University, Qingdao, China; ^2^ Department of Health Office, Qingdao Municipal Hospital, Qingdao, China; ^3^ Department of Thoracic Surgery, Qingdao Municipal Hospital, Qingdao, China; ^4^ Department of Respiratory Medicine, Qingdao Municipal Hospital, Qingdao, China

**Keywords:** ABI3BP, lung tumor, immunity infiltration, prognosis biomarker, diagnostic

## Abstract

**Background:** The primary factor of cancer mortality is lung tumor. ABI3BP gene encodes an extracellular matrix bind protein associated to multiplication and derivation. However, the prognosis score of ABI3BP for lung tumor and its relation with immunity cellular infiltration for lung tumor have not been reported.

**Methods:** Public repository systems (Timer, GEPIA, TCGA, HPA) were utilized to explore expression of ABI3BP for lung tumor, and explored the relation of ABI3BP and clinicopathological parameters. TCGA information set was utilized for cox analysis for data with one or more variables of ABI3BP for lung tumor. STRING was utilized to explore ABI3BP regulatory networks. GO/KEGG enrichment analysis as well as enrichment analysis of gene sets were carried out for ABI3BP co-expression *via* R package. And finally we explored the relation of expression of ABI3BP and lung tumor immunity invasion, exploring the influence of ABI3BP level of expression on immunotreatment and whether immunity invasion would affect the prognosis of patients with lung tumor.

**Results:** ABI3BP is downregulated in LUAD and LUSC, and associated to lung tumor phase and prognosis. Univariate and multivariate cox regression showed that ABI3BP was an independent prognostic factor in patients with lung tumors. The extracellular matrix protein-coding gene and the ABI3BP-related gene were intersected to obtain 10 hub genes. On the basis of GO/KEGG enrichment analysis, hub genes are closely associated to immunity-associated pathways including T cell receptor signaling pathway, immune response−activating cell surface receptor signaling pathway. Finally, the expression of ABI3BP is closely related to immune cell infiltration and immune cell marker set, and the expression of ABI3BP can help predict the therapeutic effect of immune checkpoint inhibitors and improve the prognosis of patients.

**Conclusion:** ABI3BP could be a new target for lung tumor that could be utilized as a diagnostic and therapeutic tool.

## Introduction

Cancer death is primarily caused by lung tumors, which are the second most common type of cancer (Sung et al., 2021). The treatment of lung tumor mainly includes surgical resection, chemotreatment, radio-treatment, targeted treatment and immunotreatment. Although the detection and therapy for lung tumor have improved, in the five-year follow-up period, the OS rate is still lower. This makes it imperative to understand the molecular processes involved in lung cancer as well as explore new biomarkers, which are essential for optimistic prognosis.

As one of the main components of TME (the tumor microenvironment), ECM (extracellular matrix) plays an important role in the process of tumor proliferation and invasion, including providing mechanical support, cell adhesion, growth factors and intercellular communication (Walker et al., 2018). On the one hand, ECM acts as a natural barrier against tumor metastasis, controlling the proliferation, differentiation and metastasis of tumor cells. On the other hand, remodeled ECM may lead to high proliferation, low differentiation, inhibition of apoptosis, invasion and metastasis of tumor cells (Huang et al., 2021). Therefore, a comprehensive understanding of the dysregulation of ECM in TME will contribute to the discovery of promising cancer therapeutic targets. ABI family member 3 bind protein is an extracellular matrix protein related with multiplication and derivation (Delfin et al., 2019). It is expressed and localized in the cytoplasm of multiple organs, including cardiovascular, kidneys, lungs, brains, pancreatic, placenta, skeletal muscle, and bones, and has important health and disease roles (Matsuda et al., 2001). It has been documented that the level of ABI3BP among patients having heart failure in dilated cardiomyopathy is significantly reduced (DeAguero et al., 2017), and ABI3BP controls the multiplication and derivation of cardiac progenitor cells. At the same time, the decreased expression of ABI3BP has a protection impact on cigarette smoke-induced emphysema (Radder et al., 2017). Moreover, single nucleotide polymorphisms in the ABI3BP gene were strongly related with suicide attempts (Perlis et al., 2010; Kimbrel et al., 2018). Expression of ABI3BP of mRNA was upregulated among patients having early preeclampsia in comparison with the control group matched with late preeclampsia and gestational age (Nevalainen et al., 2017). Notably, ABI3BP is indispensable in the development of human tumorigenesis as a new diagnostic target. Studies have proven that downregulates and inhibits proliferation, activity, migration and invasion of esophageal cancer cells (Cai et al., 2020). ABI3BP inhibits tumor growth by promoting aging and inhibiting invasion in thyroid cancer (Latini et al., 2008). ABI3BP acts as a gene that suppresses tumor growth during the growth and metastasis of gallbladder carcinoma at the cellular level (Lin et al., 2019). However, the biological role of ABI3BP in lung tumor has not been studied.

This is the first comprehensive investigation of the connection between ABI3BP and the development of lung tumor. This study utilized TCGA, GEPIA, UALCAN, HPA, Genecards, TIMER, TISIDB, STRING, Kaplan Meier plotter statistics, The Cancer Immunome Atlas, and R package to explore the role of ABI3BP in lung cancer. First, we analyzed the expression level of ABI3BP in lung tumors and its relationship with prognosis and clinicopathological features. We then examined gene networks that are related and functionally similar to ABI3BP expression and their biological roles. Finally, we explored the effect of ABI3BP expression on TME and immunotherapy response. Our results demonstrate the importance of ABI3BP in determining lung tumor prognosis, and that the expression of ABI3BP may influence lung tumor progression by modulating immune-related processes.

## Materials and methods

### The cancer genome atlas (TCGA)

From the Cancer Genome Atlas repository (https://portal.gdc.cancer.gov/) download patients’ RNA expression spectrums with lung tumor (594 patients with LUAD: 535 tumor cases and 59 regular cases. Relevant clinical information of 522 cases were acquired). There were 551 patients with LUSC: 502 tumor cases and 49 regular cases. Relevant clinical information were acquired for 504 cases. We analyzed the prognosis and diagnostic importance of expression of ABI3BP for lung tumor *via* cox model on the basis of relevant clinical information.

### Gene expression profile interaction analysis (GEPIA)

A repository called the Gene Expression Profile Interaction Analysis (http://gepia.cancer-pku.cn/) had been utilized for the purpose of examining information about RNA sequence expression from 8,587 regular and 9,736 cancerous cell specimens from TCGA projects (Tang et al., 2017). We analyzed expression of ABI3BP in LUAD and LUSC *via* GEPIA.

### The University of Alabama at Birmingham cancer information analysis portal (UALCAN)

A web-based interactive tool for the analysis of tumor-omics information is UALCAN (http://ualcan.path.uab.edu/index.html) (Chandrashekar et al., 2022). The UALCAN repository was applied to identify the relation of the expression of ABI3BP.

### Human protein atlas (HPA)

HPA (https://www.proteinatlas.org/) is an accessible repository designed in order to identify all human proteins within cells, tissues, and organs using different omics techniques (Uhlen et al., 2017). We utilized HPA to explore the protein level of expressions of ABI3BP in regular and lung cancerous cells.

### The kaplan meier plotter

The Kaplan Meier Plotter (https://kmplot.com/) is an Internet repository, which could evaluate rate of survivals at different levels of genes in 21 cancer types (Lanczky and Győrffy, 2021). The Kaplan Meier database was used to explore the relationship between ABI3BP expression and patient prognosis under immune cell infiltration.

### Gene ontology function and kyoto encyclopedia of genes and genomes pathway enrichment analysis

Firstly, TCGA-LUAD as well as TCGA-LUSC information sets were utilized to explore the co-expressed genes related with expression of ABI3BP. The filter condition of correlation coefficient was set as 0.6 (corFilter is equal to 0.6), and that of correlation test *p* value was set as 0.001 (pFilter = is equal to 0.001). GO analysis is an efficient bio-informatics approach for analyzing gene-associated biological processes, cell composition, and molecular function of hub genes. GO and KEGG analysis was utilized to explore the underlying mechanism of hub genes. GO & KEGG are executed by “ClusterProfiler” of R packages.

### GeneCards

GeneCards (https://www.genecards.org/) is a searchable comprehensive database that automatically integrates data (including genomics, transcriptomics, proteomics, etc.) from about 125 web-sourced genes (Stelzer et al., 2016). Extracellular matrix protein genes were downloaded from GeneCards website, and a total of 16,144 genes were obtained. A total of 3,525 related genes with correlation coefficient greater than 10 were screened.

### TIMER

TIMER (https://cistrome.shinyapps.io/timer/) is a web portal that provides interactive features to explore immunity infiltration at different levels (Li et al., 2017). In this research, TIMER was applied to assess expression of ABI3BP among various tumors, spearman correlation was applied to explore the correlation for ABI3BP and immunity cell invasion and immunity cellular markers, and the relation of CNV and immunity cell invasion was analyzed.

### TISIDB

TISIDB repository (http://cis.hku.hk/TISIDB/) is a portal for cancer and immune system interactions, integrates heterogeneous types of information (Ru et al., 2019). In This research, we utilized TISIDB to explore the relation of ABI3BP and tumor-infiltrating lymphocytes, immunity modulators, and chemokines.

### The cancer immunome atlas (TCIA)

TCIA (https://tcia.at/home) provides comprehensive immunogenomic analysis outcomes of NGS information from TCGA and other sources for 20 solid tumors (Van Allen et al., 2015). In order to obtain the immunity cell percentage score for immunity treatment, we utilized TCIA to download the information from patients with LUAD and LUSC. IPS reflects a patient’s ability to respond to ICIs, and the IPS score ranges from 0 to 10, where greater immunogenicity is favorably related with greater values.

### STRING

STRING repository (https://cn.string-db.org/) provides information on 24′584′628 proteins from 5,090 organisms (Szklarczyk et al., 2021). We constructed protein networks of ABI3BP-related genes by String.

### Data analysis

Statistical tests were performed using R 4.1.3. Univariate and multivariate analyses were performed using Cox regression model. Spearman correlation coefficient was used to evaluate the correlation of gene expression. *p* < 0.05 defined as statistically significant.

## Results

### Expression of ABI3BP in lung tumor

**FIGURE 1:**
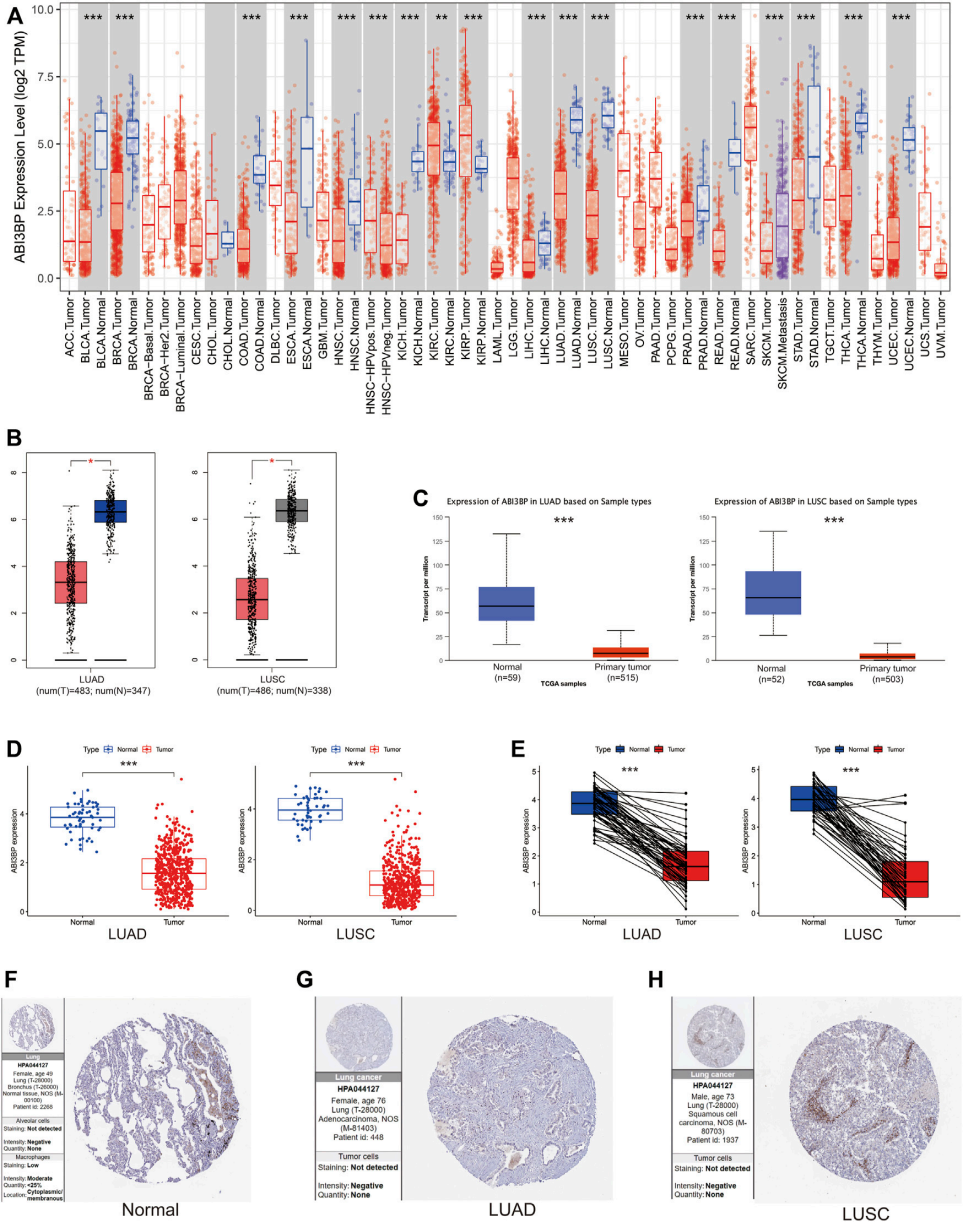
Expression of ABI3BP in lung tumors. **(A)** The expression of ABI3BP in different types of cancer was analyzed using TIMER repository. **(B)** The expression of ABI3BP in regular and lung cancerous tissues was examined using GEPIA. **(C)** The expression of ABI3BP in lung cancerous tissues and normal tissues was analyzed using UALCAN. **(D)** The expression of ABI3BP in lung tumor and regular lung tissue was analyzed using TCGA database. **(E)** Paired expression of ABI3BP in lung tumors was analyzed using TCGA. The expression level of ABI3BP in normal lung tissue **(F)**, LUAD tissue **(G)**, and LUSC tissue **(H)** was detected in the HPA database. *, *p* < 0.05 **, *p* < 0.01 ***, *p* < 0.001.

Using the repository of the TIMER database the expression of mRNA of ABI3BP in human cancer had been examined (Figure 1A). As a result of the study, lower expression of ABI3BP was observed in the following tumor types: Bladder Urothelial Carcinoma, Breast Invasive Carcinoma, Colon Adenocarcinoma, Esophageal Carcinoma, Head and Neck Squamous Cell Carcinoma, Kidney Chromophobe, Liver hepatocellular carcinoma, Lung adenocarcinoma, Lung squamous cell carcinoma, Prostate adenocarcinoma, Rectum adenocarcinoma, Stomach adenocarcinoma, Thyroid carcinoma, Uterine Corpus Endometrial Carcinomax. In contrast, expression of ABI3BP was higher in Kidney Renal Clear Cell Carcinoma and Kidney Renal Papillary Cell Carcinoma (Figure 1A). As compared to regular lung tissues, expression of ABI3BP was reduced among LUAD and LUSC tissues in the GEPIA and UALCAN repository systems (Figures 1B, C). TCGA was then utilized to evaluate expression of ABI3BP in tumor samples and regular tissues. Expression of ABI3BP was considerably lower in LUAD and LUSC tissues compared to regular specimens (Figure 1D). ABI3BP was considerably downregulated in 57 LUAD-paired tissue groups and 49 LUSC tumor groups (Figure 1E). We analyzed expression of ABI3BP in lung tumor and regular lung tissue using the Human Protein Atlas repository. The amount of expression of ABI3BP in regular lung tissues was substantially greater than in lung cancer tissues (Figures 1F–H). These outcomes imply that ABI3BP over-expression may prevent the development of lung cancer.

### Relationship between ABI3BP expression and clinicopathological features in lung tumor patients

**FIGURE 2:**
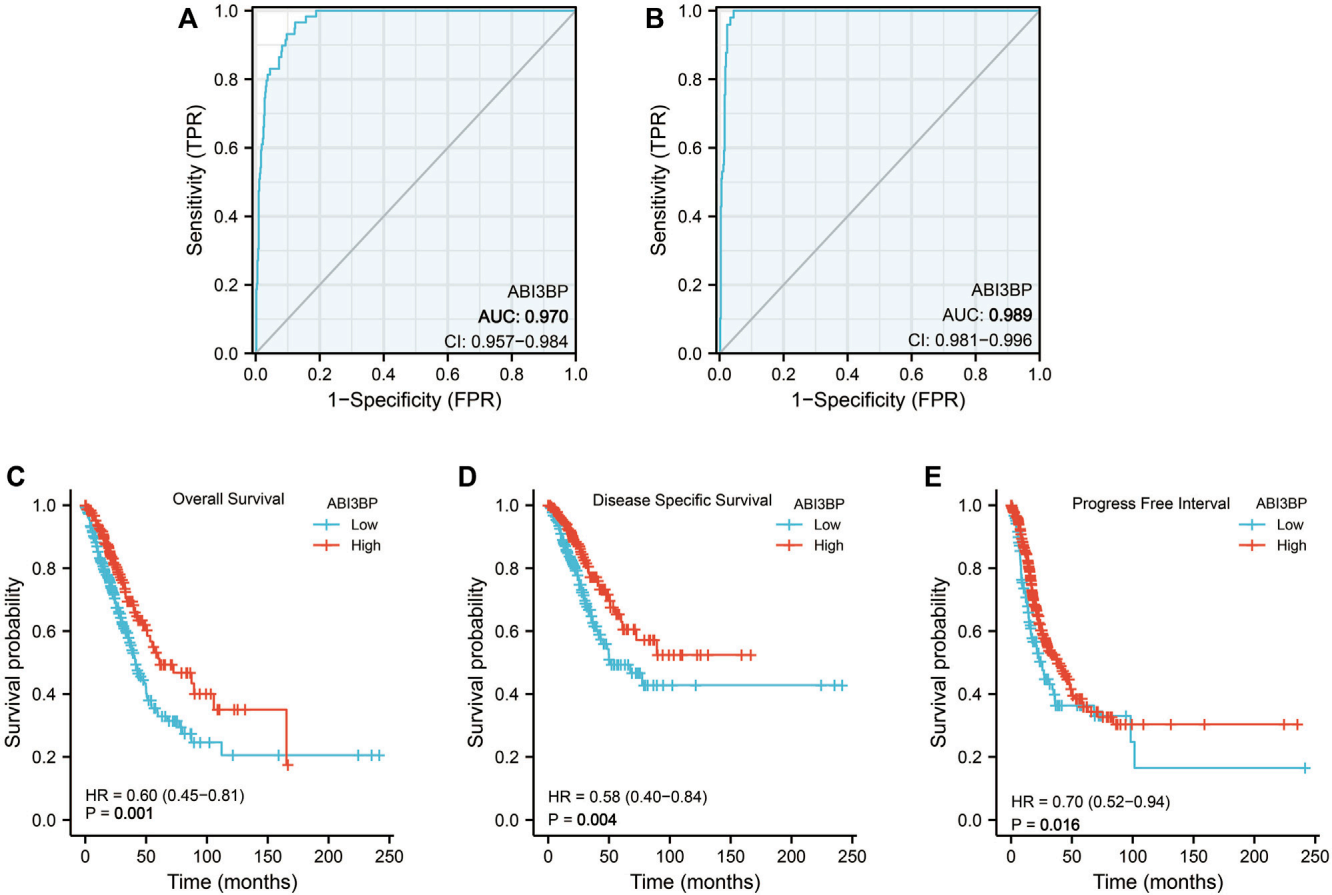
Diagnostic and prognostic value of ABI3BP. **(A, B)**Diagnostic ROC curve of ABI3BP in lung cancer. **(C–E)** Prognostic K-M curve of ABI3BP in lung cancer.

First, we explore the diagnostic significance and prognostic value of ABI3BP for lung cancer. The “pROC” package was used for ROC analysis, and it could be seen that the area under the diagnostic ROC curve was greater than 0.9, which indicated that ABI3BP had a good diagnostic value for lung cancer (Figures 2A, B). Low expression of ABI3BP is associated with poor prognosis for OS, DSS, and PFS (Figures 2C–E). These results suggest that ABI3BP is a promising diagnostic and prognostic biomarker for lung tumors.

**FIGURE 3:**
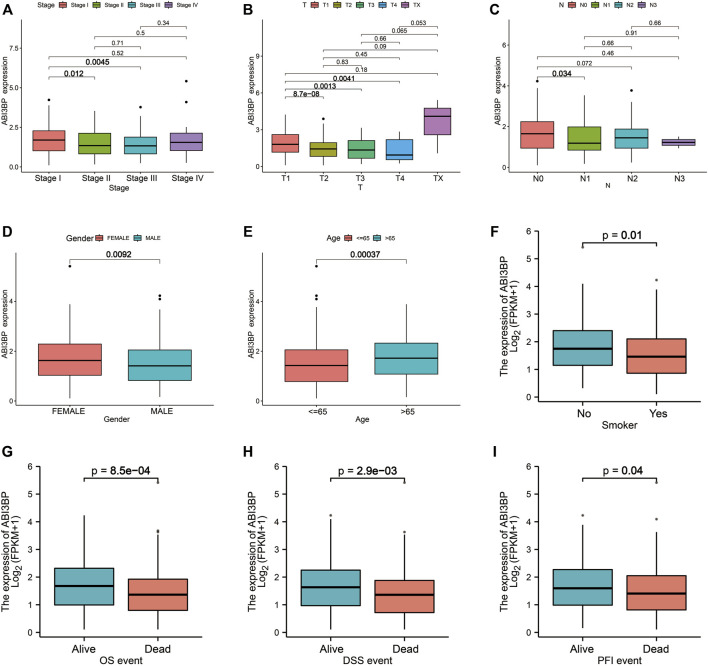
The relation of ABI3BP and clinical features of patients with lung tumor. The relationship between mRNA expression levels of ABI3BP and the stages **(A)**, T stages **(B)**, N stages **(C)**, sex **(D)**, age **(E)**, smoking history **(F)**, OS events **(G)**, DSS events **(H)**, and PFS events **(I)**. *, *p* < 0.05 **, *p* < 0.01 ***, *p* < 0.001.

Then, we collected clinical data of lung tumor patients in the TCGA database and analyzed the relationship between ABI3BP expression and clinicopathological parameters. For clinical stage, mRNA expression levels of ABI3BP gradually decrease in stage I, II, and III as lung cancer progresses (Figure 3A). Similarly, with the progression of T stage and N stage, the mRNA expression level of ABI3BP also gradually decreased (Figures 3B, C). In different gender and age groups, it can be seen that the expression of ABI3BP in female patients is significantly higher than that in male patients (Figure 3D), and the expression of ABI3BP in the elderly group (>65 years old) is higher than in the ≤65-year-old group (Figure 3E). Smoking was also an important factor affecting the level of ABI3BP, and the expression level of ABI3BP was higher in the non-smoking group than in the smoking group (Figure 3F). During OS, DSS, and PFS events, ABI3BP levels were higher in the alive group than in the death group (Figures 3G–I). In the previous study, we found that ABI3BP showed significant differences in prognosis and clinical data, so we further explored whether ABI3BP can be an independent prognostic factor for lung cancer patients.

**FIGURE 4:**
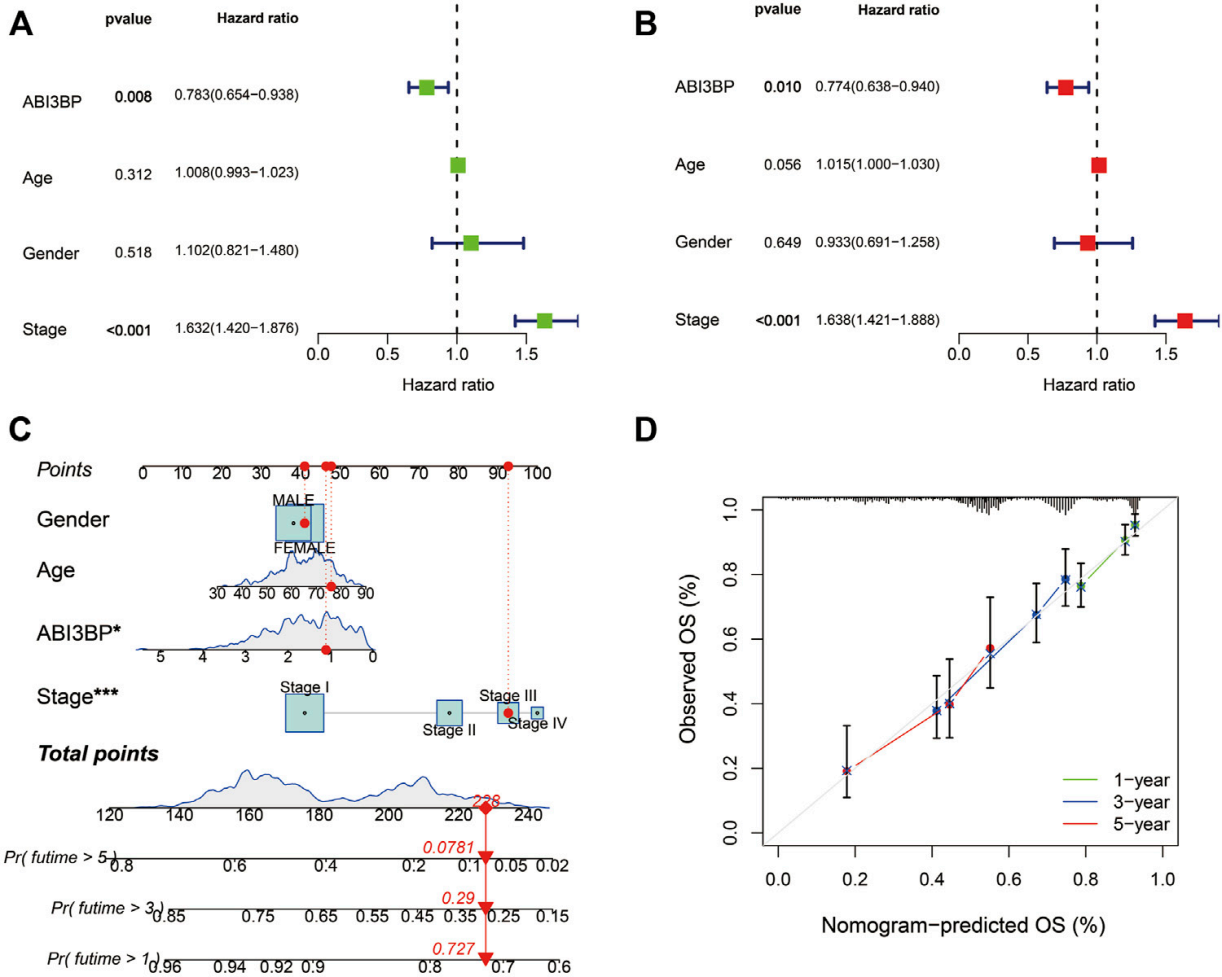
Multiple Cox regression and Nomogram construction in lung cancer patients. **(A, B)** Univariate and multivariate regression analysis of ABI3BP. **(C, D)** Construction and verification of nomogram for predicting 1-year, 3-year, and 5-year OS in lung cancer patients based on ABI3BP expression.

Finally, TCGA information sets were split into two groups on the basis of ABI3BP median expression, and the cox percentage risk regression model was utilized to explore prognosis factors. The Forest diagram showed the univariate and multivariate cox models of ABI3BP. Univariate cox regression analysis showed that low expression of ABI3BP and elevated clinical stage were significantly associated with poor OS. In addition, multivariate regression analysis further confirmed that ABI3BP expression and Stage were independent prognostic factors for OS in lung cancer patients (Figures 4A, B). The calibration curve provides an ideal prediction of the nomogram of clinical outcomes at 1-year, 3-year, and 5-year (Figures 4C, D). Based on the above data, ABI3BP can be used as a useful biomarker for predicting lung cancer OS.

### ABI3BP co-expression network and biological roles for lung tumor

**FIGURE 5:**
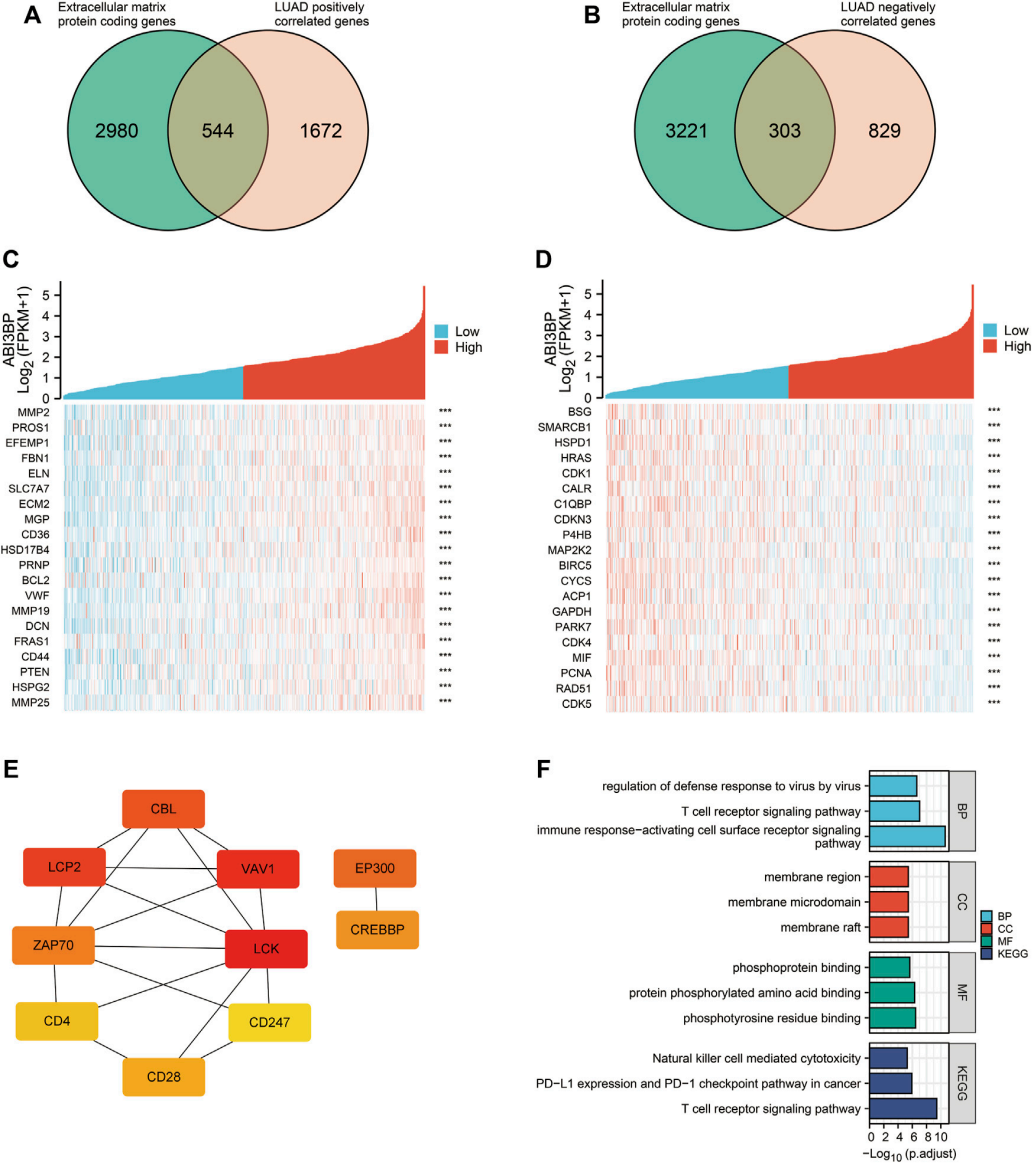
Analysis of co-expression network of ABI3BP gene in LUAD. **(A)** Venn diagram of genes intersection of the Extracellular matrix protein coding genes and ABI3BP positively correlated genes. **(B)** Venn diagram of genes intersection of the Extracellular matrix protein coding genes and ABI3BP negatively correlated genes. The top 20 genes positively **(C)** and negatively **(D)** related with ABI3BP. **(E)** Identification of the hub genes. **(F)** GO & KEGG enrichment analysis of the hub genes in LUAD.

In previous studies, we explored the relationship between ABI3BP and clinical features and found that ABI3BP expression levels correlated with patient outcomes. After further discussion, ABI3BP may become a new tumor marker. Therefore, in the next study, we further construct the protein network of ABI3BP to study the biological role of gene sets with similar functions and effects to ABI3BP. We screened out genes associated with ABI3BP in the TCGA database and intersected with the set of extracellular matrix protein-coding genes in GeneCards (Figures 5A, B; Supplementary Figure S1A, B). The heat map shows the top 20 genes with the highest positive and negative correlation with ABI3BP (Figures 5C, D; Supplementary Figure S1C, D). In the String database, the protein network of positively correlated intersection genes in LUAD and LUSC was constructed (Supplementary Figure S2A, B), then we used the Cytoscape plugin “cytoHubba” to screen out 10 hub genes respectively (Figure 5E; Supplementary Figure S1E). To gain insight into the biological significance of the hub genes, we performed GO and KEGG enrichment analyses on the hub genes. GO enrichment analysis showed that the hub genes was mainly involved in biological processes such as “T cell receptor signaling pathway”, “immune response−activating cell surface receptor signaling pathway”, and “regulation of defense response to virus by virus” in LUAD (Figure 5F). KEGG analysis showed that the hub genes are mainly involved in “Natural killer cell mediated cytotoxicity”, “PD−L1 expression and PD−1 checkpoint pathway in cancer”, and “T cell receptor signaling pathway” in LUAD (Figure 5F). At the same time, GO and KEGG analysis found that hub genes are mainly involved in “respiratory burst”, “superoxide−generating NADPH oxidase activity”, and “Leukocyte transendothelial migration” in LUSC (Supplementary Figure S1F). The above suggests that the hub genes may be involved in immune-related processes within tumor cells. So we further explored the relationship between the hub genes and immune infiltration.

### Correlation of the hub genes with immune cell infiltration

**FIGURE 6:**
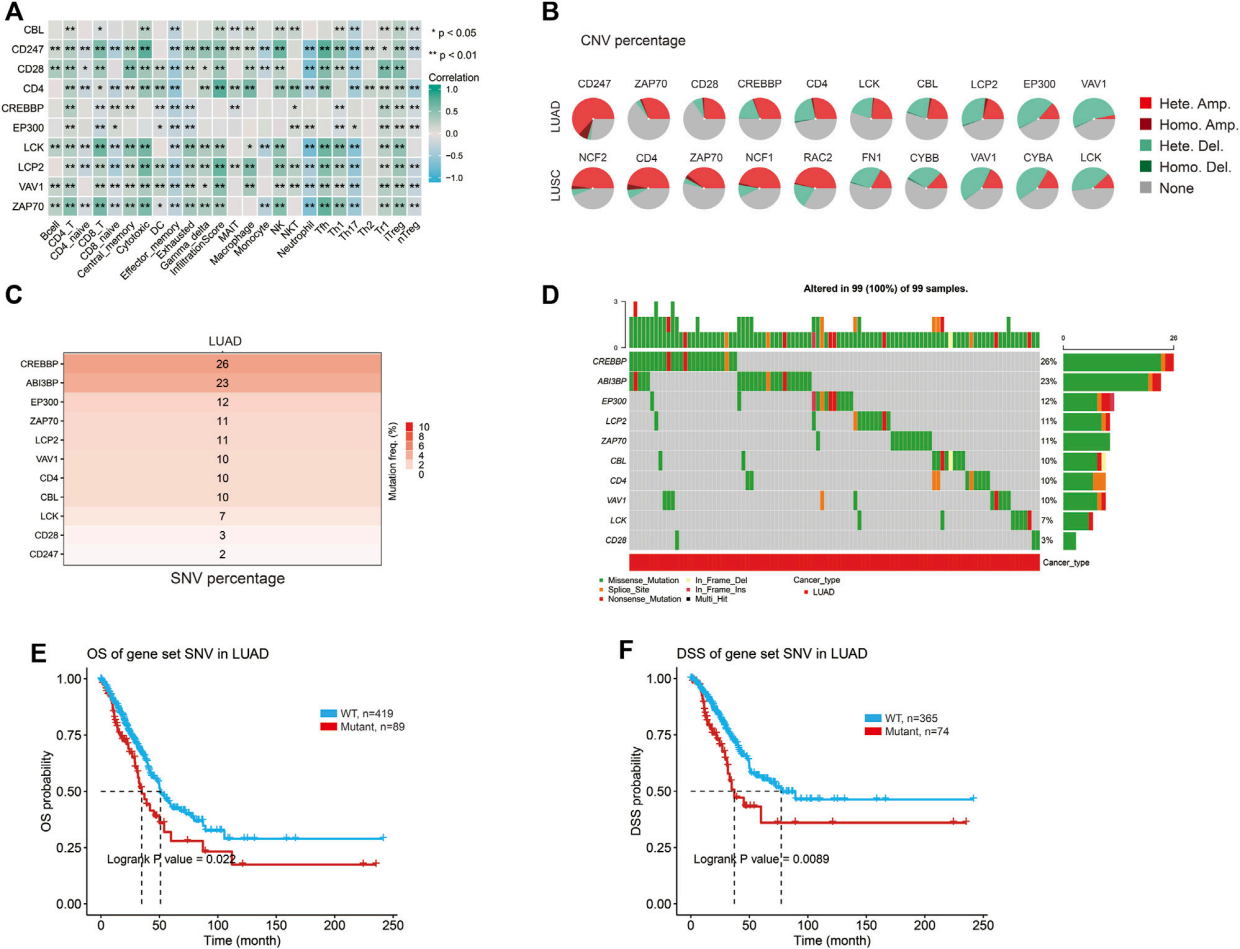
The specific mutation of the hub genes. **(A)** Relationship between the hub genes and immune cells. **(B)** The CNV percentage of hub genes in lung cancer. **(C)** SNV mutation frequency of hub genes in LUAD. **(D)** Oncoplot provides the situation of the SNV of the hub genes in LUAD. OS **(E)** and DSS **(F)** of hub genes SNV in LUAD.

The abundance of 24 immune cell types was estimated based on the hub gene set characteristics, and it was found that hub genes were related to immune cells, and most of them were positively correlated (Figure 6A). Harmful mutations of genes may affect the immune infiltration of tumor cells to promote cancer progression, so we further explored the specific mutations of hub genes. The pie chart summarizes the types and proportions of CNV (Copy Number Variation) mutations in the hub genes in LUAD and LUSC (Figure 6B). The highest percentage of copy number heterozygous amplification in LUAD was CD247 and in LUSC was NCF2. The sample percentage of copy number heterozygous deletion was the highest in VAV1 in LUAD, and the highest in CYBA and VAV1 in LUSC (Figure 6B). The heat map showed that the frequency of SNV (Single Nucleotide Variant) deleterious mutations in CREBBP was the highest in LUAD, accounting for 26% (Figure 6C). Among LUSC, FN1 had the highest frequency of SNV mutation, accounting for 38% (Supplementary Figure S2C). The oncoplot provides the SNV profile of the top 10 mutated genes in the hub gene set for lung cancer (Figure 6D; Supplementary Figure S2D). Kaplan-Meier curves were used to show the effect of the combined SNV status of the hub gene set on prognosis. And, the results showed a higher risk of death in the mutant group (Figures 6E, F). Therefore, it can be concluded that the hub genes related to ABI3BP may be involved in immune cell infiltration through mutation, thus affecting the progression of lung cancer.

### Relationship between ABI3BP and immunoinfiltration

**FIGURE 7:**
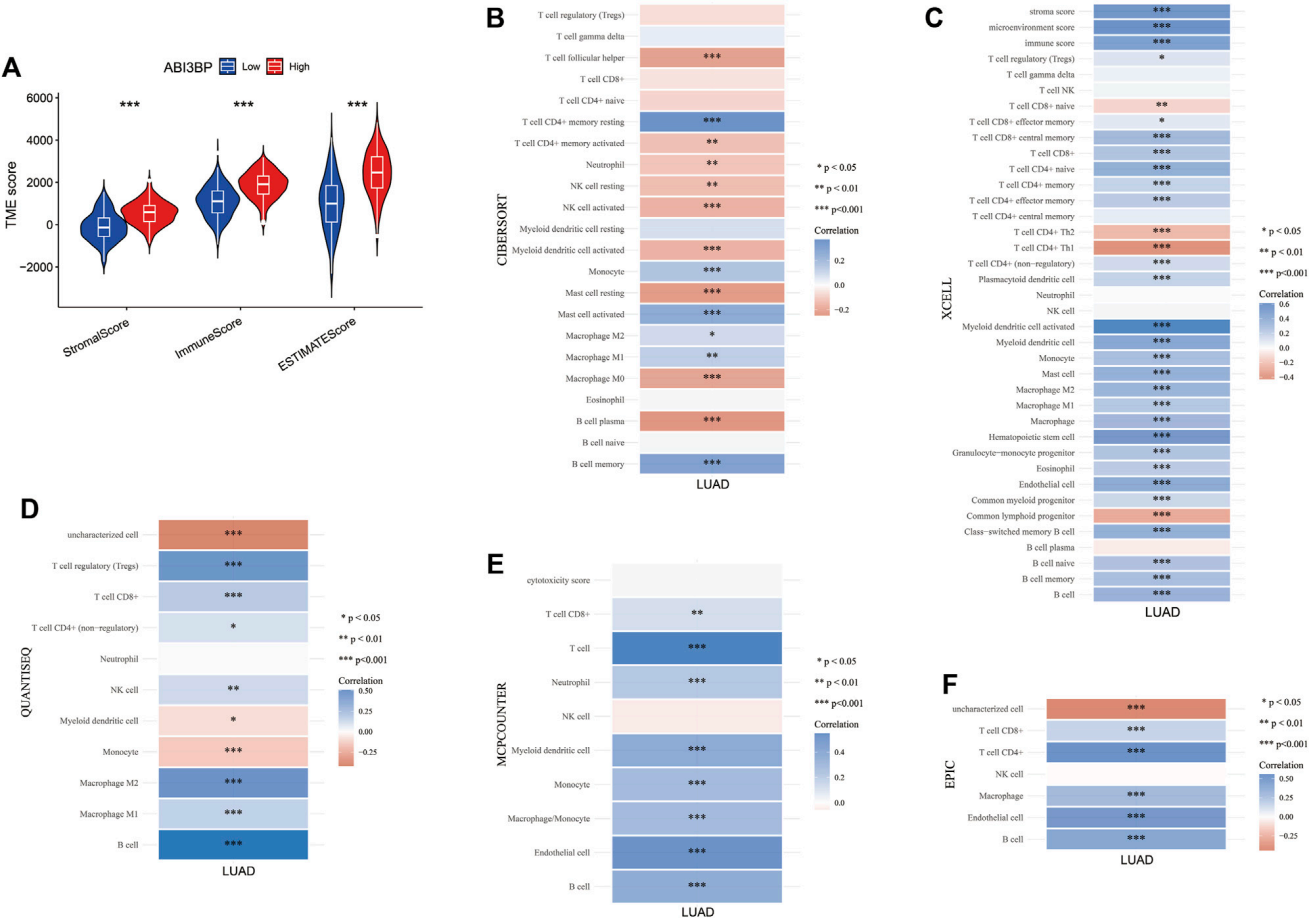
Relationship between ABI3BP and immunoinfiltration in LUAD. **(A)** TME score of ABI3BP. CIBERSORT **(B)**, XCELL **(C)**, QUANTISEQ **(D)**, MCPCOUNTER **(E)**, and EPIC **(F)** analysis between ABI3BP and tumor immune cells in LUAD.

**FIGURE 8:**
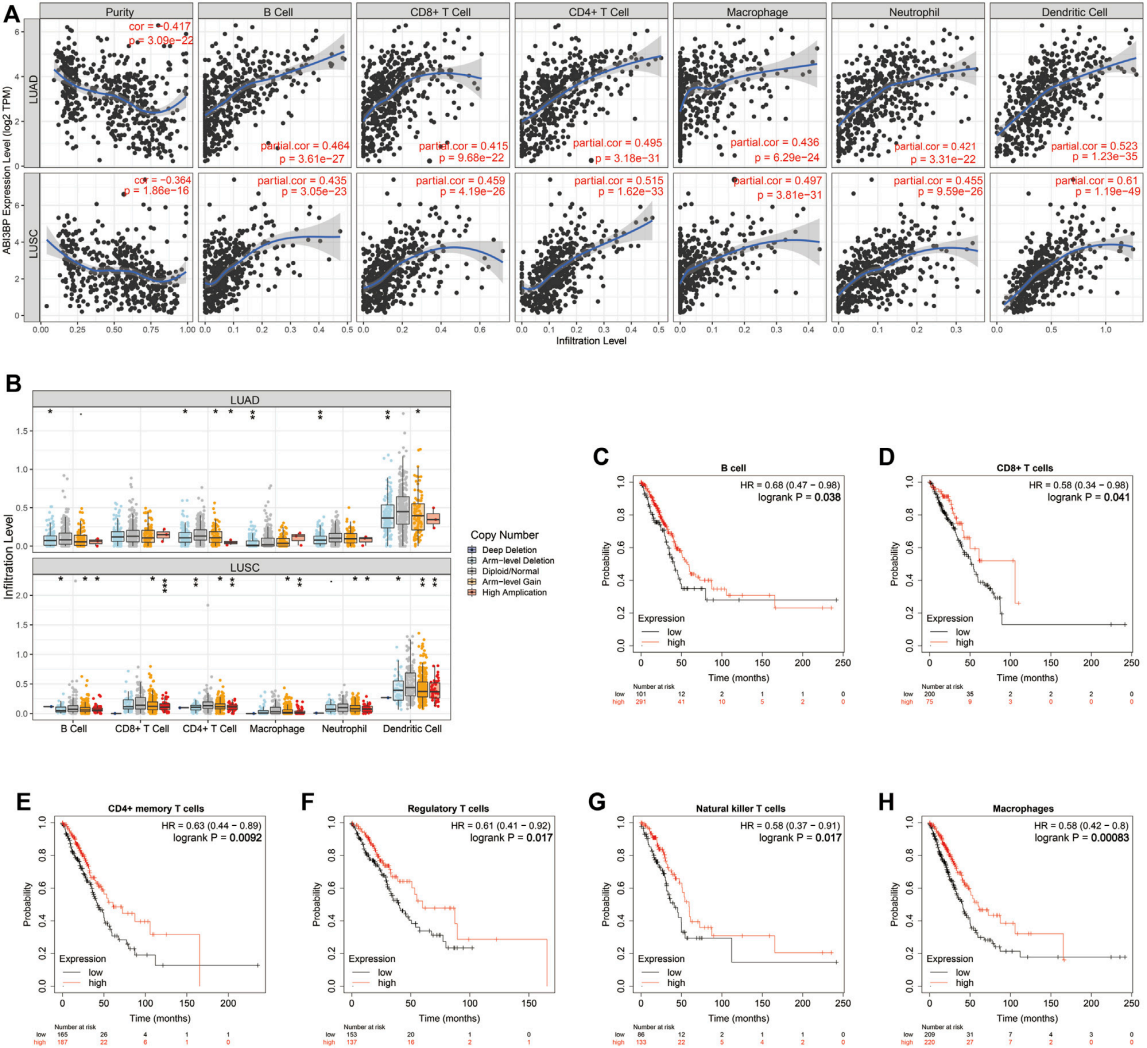
Relation of expression of ABI3BP and immune system infiltrating cells for lung tumor on the basis of TIMER repository. **(A)** The TIMER algorithm analyzes the relationship between ABI3BP and immune cell infiltration in LUAD and LUSC; **(B)** ABI3BP CNV in LUAD and LUSC affected the infiltration levels of B-cell, CD8^+^ T-cell, CD4^+^ T-cell, Macrophages, Neutrophils and Dendritic cells; **(C–H)** Relationship between expression of ABI3BP and prognosis in patients under immune cell infiltration. *p < 0.05; **p < 0.01; ***p < 0.001.

Next, we explored the potential molecular mechanism of ABI3BP in lung cancer immunoinfiltration. First, we performed TME score on patient data in the TCGA database, including the StromalScore, the ImmuneScore, and the ESTIMATEScore. It was found that the TME score of the high ABI3BP expression group was significantly higher than that of the low ABI3BP expression group (Figure 7A; Supplementary Figure S3A). Next, we used the “immunedeconv” package to demonstrate the relationship between ABI3BP and tumor immune cells in LUAD and LUSC, respectively. CIBERSORT algorithm shows that ABI3BP is significantly positively correlated with T-cell CD4^+^ memory resting and B cell memory in both LUAD and LUSC (Figure 7B; Supplementary Figure S3B). The results of XCELL algorithm showed that ABI3BP was positively correlated with the Myeloid dendritic cell activated in LUAD and LUSC (Figure 7C; Supplementary Figure S3C). Then, QUANTISEQ analysis showed a significant positive correlation between ABI3BP and T cell regulation (Tregs) in lung cancer (Figure 7D; Supplementary Figure S3D). In MCPCOUNTER analysis, T cell, Endothelial cell, and B cell were positively correlated with ABI3BP (Figure 7E; Supplementary Figure S3E). The EPIC analysis also showed a positive correlation between ABI3BP and Macrophage, Endothelial cell and B cell (Figure 7F; Supplementary Figure S3F). Finally, TIMER database analysis showed that ABI3BP had the highest correlation with dendritic cell cells in lung cancer (Figure 8A). In addition, we used the TIMER and GEPIA repository systems to assess the relationship between expression of ABI3BP and immune markers in several sub-types of TIICs for lung malignanT cell. B cell, CD8^+^ T cell, T cell follicular helpers, Th1 cells, Th2 cells, Th17 cells, Treg cells, T cell exhaustion, Macrophages M1, Macrophages M2, Tumor-Associated Macrophages, Monocytes, Natural Killer cells, Neutrophils, and Dendritic cells express ABI3BP to varying degrees (Table 1). Box plots showed that CNV mutation in ABI3BP significantly reduced the degree of immune cell infiltration (Figure 8B). Due to the significant relation of expression of ABI3BP and immune cell infiltration and prognosis of lung tumor, we then examined whether its expression affects lung cancer prognosis due to immunity invasion. Prognosis analysis was conducted on the basis of the level of expression of ABI3BP in associated immunity cell subsets. In the state of B cell, CD8^+^ T cell, CD4^+^ memory T cell, Regulatory T -cell, Natural killer cells, and macrophages enrichment, the higher the expression level of ABI3BP, the better the prognosis (Figures 8C–H). Above, high ABI3BP expression affected the level of invasion of partly immune cells in tumors, suggesting that patients had a good prognosis.

**TABLE 1:** Correlation analysis between ABI3BP and immune cell marker gene in TIMER and GEPIA.

Description	Gene markers	LUAD						LUSC					
		TIMER				GEPIA		TIMER				GEPIA	
		None		Purity		Tumor		None		Purity		Tumor	
		rho	*p*	rho	*p*	rho	*p*	rho	*p*	rho	*p*	rho	*p*
B-cell	CD19	0.495	**3.87e-33**	0.374	**8.35e-18**	0.46	**2.8e-26**	0.588	**6.98e-48**	0.499	**1.82e-31**	0.53	**2.8e-36**
	MS4A1	0.626	**0e+00**	0.529	**5.93e-37**	0.59	**5.7e-47**	0.657	**3.74e-63**	0.589	**8.62e-46**	0.61	**6.6e-51**
	CD79A	0.424	**0e+00**	0.297	**1.81e-11**	0.4	**5.5e-20**	0.559	**1.89e-42**	0.46	**2.45e-26**	0.52	**1.1e-35**
CD8^+^ T-cell	CD8A	0.433	**0e+00**	0.31	**1.95e-12**	0.42	**1.5e-22**	0.547	**1.77e-40**	0.485	**1.75e-29**	0.51	**4.5e-34**
	CD8B	0.34	**2.8e-15**	0.235	**1.35e-07**	0.33	**6.8e-14**	0.477	**7.67e-30**	0.448	**6.22e-25**	0.44	**6.2e-25**
	IL2RA	0.384	**1.69e-19**	0.264	**2.47e-09**	0.42	**2.5e-22**	0.594	**0e+00**	0.519	**2.61e-34**	0.59	**1.2e-46**
Tfh cell	BCL6	0.196	**7.73e-06**	0.204	**4.86e-06**	0.27	**2.2e-09**	0.051	2.52e-01	0.105	**2.17e-02**	0.11	**0.02**
	CXCR5	0.596	**0e+00**	0.495	**7.66e-32**	0.11	**0.015**	0.681	**1.81e-69**	0.621	**4.00e-52**	0.23	**1.7e-07**
	ICOS	0.557	**2.58e-43**	0.437	**1.89e-24**	0.55	**3.4e-40**	0.639	**9.52e-59**	0.568	**4.14e-42**	0.6	**1.6e-49**
Th1 cell	IL12RB1	0.565	**9.67e-45**	0.454	**2.03e-26**	0.59	**1.6e-46**	0.657	**0e+00**	0.587	**1.92e-45**	0.65	**5.2e-59**
	STAT1	0.27	**5.69e-10**	0.173	**1.08e-04**	0.31	**5.3e-12**	0.319	**3.56e-13**	0.261	**7.28e-09**	0.31	**5.8e-12**
	CCR5	0.597	**0e+00**	0.49	**4.39e-31**	0.62	**5.32e-53**	0.69	**0e+00**	0.634	**4.56e-55**	0.69	**8.1e-69**
Th2 cell	CCR4	0.668	**6.25e-68**	0.607	**7.17e-51**	0.7	**8.9e-72**	0.732	**4.17e-85**	0.681	**3.26e-66**	0.74	**3.2e-84**
	STAT6	0.283	**7.49e-11**	0.324	**1.52e-13**	0.32	**4.2e-13**	0.17	**1.35e-04**	0.183	**5.76e-05**	0.17	**0.00011**
	HAVCR1	0.036	4.09e-01	0.042	3.48e-01	0.092	**0.043**	0.309	**1.52e-12**	0.302	**1.60e-11**	0.27	**2.5e-09**
Th17 cell	IL21R	0.509	**0e+00**	0.376	**5.43e-18**	0.54	**6.9e-38**	0.668	**0e+00**	0.603	**1.39e-48**	0.67	**6.3e-64**
	IL23R	0.411	**1.86e-22**	0.383	**1.13e-18**	0.44	**1.9e-24**	0.322	**1.51e-13**	0.256	**1.49e-08**	0.3	**6.4e-12**
	CCR6	0.695	**0e+00**	0.63	**8.04e-56**	0.69	**1.2e-69**	0.699	**0e+00**	0.636	**2.10e-55**	0.66	**1.3e-62**
Treg cell	STAT5B	0.465	**5.07e-29**	0.466	**6.55e-28**	0.49	**2.9e-30**	0.294	**2.44e-11**	0.322	**5.44e-13**	0.26	**6.3e-09**
	NT5E	0.172	**8.96e-05**	0.155	**5.75e-04**	0.19	**2.9e-05**	0.19	**1.85e-05**	0.109	**1.68e-02**	0.2	**6.8e-06**
	CCR8	0.497	**2.04e-33**	0.395	**6.60e-20**	0.54	**3.1e-38**	0.631	**5.17e-57**	0.566	**8.30e-42**	0.63	**4.8e-54**
	IL7R	0.671	**0e+00**	0.594	**2.80e-48**	0.71	**1.5e-75**	0.591	**0e+00**	0.509	**8.29e-33**	0.58	**6.9e-46**
T-cell exhaustion	PDCD1	0.368	**6.1e-18**	0.227	**3.40e-07**	0.37	**2e-17**	0.584	**4.2e-47**	0.514	**1.81e-33**	0.56	**4.2e-42**
	CTLA4	0.443	**0e+00**	0.303	**6.42e-12**	0.45	**1.2e-25**	0.627	**0e+00**	0.555	**6.13e-40**	0.61	**1.8e-50**
	LAG3	0.264	**1.32e-09**	0.139	**1.99e-03**	0.25	**1.6e-08**	0.438	**0e+00**	0.375	**2.19e-17**	0.4	**1.2e-19**
	HAVCR2	0.467	**0e+00**	0.346	**2.57e-15**	0.49	**2.4e-30**	0.641	**0e+00**	0.571	**1.37e-42**	0.62	**3.5e-52**
M1 Macrophage	NOS2	0.221	**4.2e-07**	0.159	**4.05e-04**	0.25	**2.1e-08**	0.157	**4.13e-04**	0.189	**3.30e-05**	0.15	**0.00084**
	IRF5	0.336	**6.39e-15**	0.234	**1.41e-07**	0.34	**1.2e-14**	0.144	**1.24e-03**	0.122	**7.60e-03**	0.12	**0.0095**
	PTGS2	−0.067	1.27e-01	−0.069	1.27e-01	−0.053	0.25	0.124	**5.64e-03**	0.062	1.75e-01	0.15	**0.0013**
M2 Macrophage	CD163	0.476	**0e+00**	0.38	**2.33e-18**	0.44	**8.5e-25**	0.632	**0e+00**	0.567	**6.05e-42**	0.63	**1.8e-54**
	MRC1	0.552	**0e+00**	0.484	**2.77e-30**	0.59	**1.4e-47**	0.583	**6.16e-47**	0.521	**1.59e-34**	0.59	**7.9e-48**
	CD209	0.407	**0e+00**	0.301	**8.54e-12**	0.47	**2e-28**	0.464	**0e+00**	0.385	**2.43e-18**	0.49	**1.7e-30**
	MS4A4A	0.492	**0e+00**	0.391	**1.82e-19**	0.52	**2.3e-34**	0.598	**0e+00**	0.526	**2.89e-35**	0.58	**3e-45**
TAM	CCL2	0.307	**1.32e-12**	0.189	**2.40e-05**	0.34	**3.5e-14**	0.55	**0e+00**	0.493	**1.44e-30**	0.53	**1.3e-36**
	CD68	0.462	**0e+00**	0.372	**1.32e-17**	0.51	**1.9e-33**	0.5	**0e+00**	0.409	**1.24e-20**	0.49	**3.6e-31**
	IL10	0.451	**3.28e-27**	0.333	**3.20e-14**	0.45	**1.6e-25**	0.528	**2.45e-37**	0.467	**3.3e-27**	0.49	**6.7e-31**
Monocyte	CD14	0.342	**1.72e-15**	0.217	**1.10e-06**	0.38	**4.8e-18**	0.565	**0e+00**	0.47	**1.23e-27**	0.56	**1.1e-40**
	CD33	0.532	**0e+00**	0.439	**1.15e-24**	0.52	**1.6e-35**	0.644	**5.17e-60**	0.574	**3.42e-43**	0.62	**2.1e-52**
	ITGAX	0.509	**2.89e-35**	0.403	**1.00e-20**	0.51	**2.7e-33**	0.636	**0e+00**	0.557	**2.75e-40**	0.6	**2.7e-49**
Natural killer cell	B3GAT1	0.305	**1.54e-12**	0.282	**1.87e-10**	0.3	**1.2e-11**	0.482	**1.74e-30**	0.431	**5.02e-23**	0.46	**3.4e-27**
	KIR3DL1	0.156	**3.91e-04**	0.089	**4.81e-02**	0.22	**1.3e-06**	0.376	**2.91e-18**	0.329	**1.73e-13**	0.41	**1.3e-20**
	CD7	0.253	**6.19e-09**	0.12	**7.57e-03**	0.26	**6.9e-09**	0.55	**0e+00**	0.462	**1.28e-26**	0.53	**1.6e-36**
Neutrophils	FCGR3A	0.36	**2.2e-17**	0.25	**1.81e-08**	0.41	**5.7e-21**	0.584	**0e+00**	0.513	**2.49e-33**	0.58	**7.9e-45**
	CCR7	0.698	**0e+00**	0.618	**3.68e-53**	0.68	**3.9e-68**	0.717	**0e+00**	0.664	**4.45e-62**	0.69	**4.8e-70**
	CD55	0.045	3.09e-01	0.072	1.09e-01	0.059	0.19	0.223	**4.76e-07**	0.17	**1.87e-04**	0.18	**6.7e-05**
	ITGAM	0.509	**0e+00**	0.42	**1.64e-22**	0.58	**2e-44**	0.649	**0e+00**	0.589	**5.82e-46**	0.68	**4e-67**
Dendritic cell	CD1C	0.541	**2.03e-40**	0.482	**4.82e-30**	0.51	**8.8e-34**	0.537	**9.61e-39**	0.438	**9.80e-24**	0.48	**1.8e-29**
	THBD	0.431	**1.18e-24**	0.377	**4.66e-18**	0.48	**1.6e-29**	0.052	2.41e-01	0.01	8.22e-01	0.12	0.071
	NRP1	0.2	**4.91e-06**	0.174	**1.05e-04**	0.25	**1.5e-08**	0.474	**0e+00**	0.398	**1.35e-19**	0.48	**6.4e-30**

Bold values indicate *p* < 0.05.

### Relationship between ABI3BP and immunity signals

**FIGURE 9:**
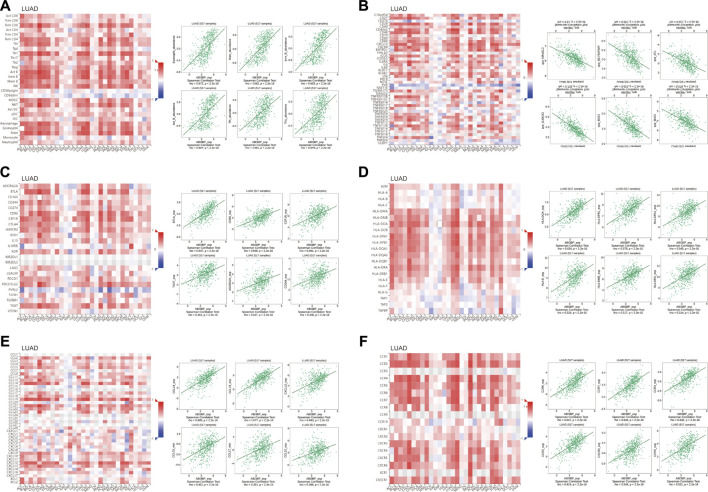
Relation of ABI3BP level of expression and lymphocytes, immunity modulators and chemokines in LUAD on the basis of TISIDB repository. **(A)** Relation of tumor-infiltrating lymphocyte (TIL) abundance and ABI3BP (6 TIL with the greatest correlation); **(B–D)** Relation of immunomodulators and ABI3BP (the six immunomodulators with the greatest correlation); **(E–F)** Relation of chemokines (or receptors) and ABI3BP [6 chemokines (or receptors) with the greatest correlation respectively].

To further our understanding of the relationship between ABI3BP and immune invasion, we used the TISIDB library to evaluate the relation of expression of ABI3BP and numerous immunity signals, including 28 tumor immunity Lymphocyte sub-types. Immunostimulants, immunoreceptors, molecules of the major histocompatibility complex, chemokines, and receptors. ABI3BP was related with tumor-infiltrating lymphocytes in LUAD and LUSC, respectively (Figure 9A; Supplementary Figure S4A). The relationship between expression of ABI3BP and immune modulators was shown in Figures 9B–D. Expression of ABI3BP was related with 20 immunosuppressive molecules, 36 immunostimulatory molecules, and 21 major histocompatibility complex molecules in LUAD (Figures 9B–D). 19 immunosuppressive molecules, 41 immunostimulatory molecules, and 21 major histocompatibility complex molecules were related with expression of ABI3BP in LUSC (Supplementary Figures S4B–D). ABI3BP was related with 28 chemokines and 15 receptors in LUAD and 30 chemokines and 16 receptors in LUSC, according to the outcomes (Figures 9E, F; Supplementary Figure S4E, F). Supplementary Tables S1 through 6 provide further information on correlation analyses. In conclusion, ABI3BP contributes significantly to the regulation of numerous immunity molecules in lung tumors, hence influencing the immunoinfiltration in tumor micro-environments.

### Expression of ABI3BP for patients with lung tumor showed promising therapeutic value

**FIGURE 10:**
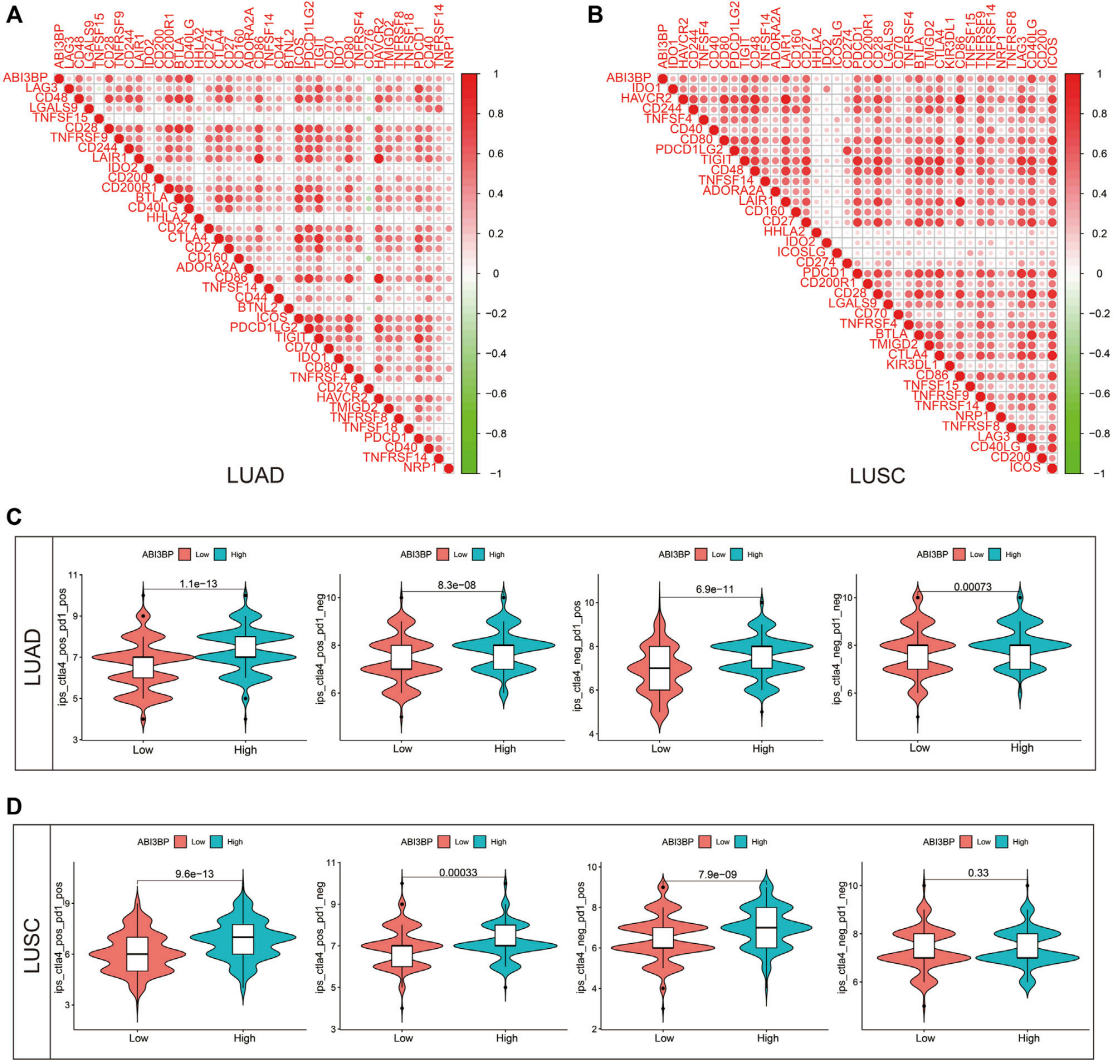
Therapeutic value of ABI3BP. **(A,B)** Relationship between ABI3BP and immune checkpoints. **(C,D)** ABI3BP and sensitivity to immunotherapy response inLUAD and LUSC.

To explore the impact of expression of ABI3BP on immuno treatment for patients with lung tumor. We initially examined the relation of ABI3BP as well as immunity checkpoint on the basis of TCGA repository. The outcomes demonstrated that ABI3BP was related with PDCD1, CTLA4 and other immunity checkpoints for lung tumor (Figures 10A, B). Previous research has shown that IPS (immunity treatment score) correlates with ICI-based immunity therapy response (immunity checkpoint inhibitors). Greater IPS is related with improved immunogenicity, suggesting a more favorable response to ICIs. Consequently, IPS was used to assess the efficiency of immunity therapy in groups with varying levels of expression of ABI3BP, and the outcomes demonstrated that immunity therapy was effective (Figure 10C). CTLA4 negative/PD1 negative, CTLA4 negative/PD1 positive, CTLA4 positive/PD1 negative, and CTLA4 positive/PD1 positive IPS were greater in the ABI3BP group with high expression of patients with LUAD than in the group with low expression (Figure 10C). CTLA4 negative/PD1 positive, CTLA4 positive/PD1 negative, and CTLA4 positive/PD1 positive IPS were all larger in the ABI3BP group with high expression than in the group with low expression in LUSC (Figure 10D). These outcomes imply that ABI3BP could be useful for predicting the success of ICI, which might give guidance for immunity therapy.

## Discussion

Among all cancer types, lung tumor has the greatest death rate worldwide (Siegel et al., 2021). The 5-year rate of survival of lung tumor remains lower than 20% despite great advances in diagnosis, targeted treatment, and immunity treatment (Chen et al., 2014). As a result, it is very important to explore other types of biomarkers for lung cancer prognosis to improve the rate of survival of patients with lung cancer. Therefore, finding markers that can predict the occurrence, development and prognosis of lung cancer and the effect of immunotherapy can help to correctly diagnose and intervene lung cancer at an early stage, improve prognosis, and reduce unnecessary adverse drug reactions. Previous studies have shown that by inhibiting ABI3BP, the long non-coding RNA, MALAT1, stimulates the proliferation of gallbladder cancer cells and prevents aging (Lin et al., 2019). MicroRNA-183 consumes ABI3BP and also promotes the growth of esophageal cancer (Cai et al., 2020). Here, for the first time, we performed bioinformatics analysis of ABI3BP using public databases (Timer, GEPIA, TCGA, and HPA) and found that lung tumors expressed significantly lower ABI3BP than conventional lung tissue. This is consistent with previous findings (Uekawa et al., 2005). Based on these results, ABI3BP may be a gene associated with lung cancer growth. Therefore, we delved into the significance between ABI3BP expression and clinical data and prognosis. It was found that the expression degree of ABI3BP was related to clinical factors such as gender, age, smoking habit, clinical stage, and lymph node metastasis status. Univariate and multivariate cox regression analysis showed that elevated ABI3BP and elevated clinical stage were independent risk factors for lung cancer patients. These findings suggest that ABI3BP can be used as a tumor suppressor gene and prognostic biomarker. In order to explore the potential molecular mechanism of ABI3BP affecting the prognosis of lung cancer patients, we screened 10 genes from GeneCards among 3,525 extracellular matrix protein-coding genes co-expressed with ABI3BP as hub genes, and deeply explored the potential mechanism of role of hub genes in lung cancer. We found that the GO/KEGG enrichment analysis of the pivot gene was deeply correlated with the immune process and positively correlated with immune cells. LCK, one of the core of LUAD pivotal genes, is an independent predictor of recurrence-free and overall survival in lung cancer patients (Chen et al., 2007), which can coordinate the tumor microenvironment with CD3E to promote immunotherapy response and survival in patients with muscle-invasive bladder cancer (Zheng et al., 2021). Another core gene, Vav1, accelerates Ras-driven lung cancer and regulates its tumor microenvironment (Shalom et al., 2022) and its mutations can cause different human oncogenic phenotypes. LCP2 is also a good prognostic biomarker for lung cancer patients (Huo et al., 2021). Most of these genes, which function similarly to ABI3BP and both play an inhibitory role in tumors, affect tumorigenesis and progression through different types of mutations. Therefore, we showed the types and proportions of CNV and SNV mutations in the pivot gene, and showed that mutations in ABI3BP and pivot genes led to poor prognosis for patients.

Further, we hypothesize that mutations in ABI3BP and related genes may act through tumor immune infiltration. First, we explored the link between the expression of ABI3BP and invasion by lung tumor immune cells. The expression of ABI3BP was significantly positively correlated with matrix scores and immunoscores, which were determined by estimation. The CIBERSORT, XCELL, QUANTISEQ, MCPCOUNTER, and EPIC methods evaluated the degree of tumor immune cell infiltration in the TCGA lung cancer dataset. We found that in lung cancer, ABI3BP was significantly positively correlated with B memory cells, CD4^+^ T memory cell rest, Tregs, B cell, T cell, CD4^+^ T, myeloid dendritic cell activation, and endothelial cells. This suggests that increased expression of ABI3BP in tumors may interfere with the tumor immune microenvironment by affecting immune cell infiltration, thereby affecting tumor progression.

Simultaneously, ABI3BP was substantially related with many immune cell marker sets for lung cancer. Moreover, ABI3BP is intimately engaged in the regulation of several immunity signaling molecules for lung tumor, including immunity stimulants, immunity suppression, MHC, chemokines, and receptors, therefore influencing the invasion of the immune system in the tumor micro-environment. The tumor micro-environment is essential for lung cancer treatment (Wood et al., 2014). Lung tumor is responsive to ICI-based immunity therapy that targets immune checkpoint blocking molecules, including Programmed cell death 1, programmed cell death lig and 1, and CTLA4 (Suresh et al., 2018; Zhou et al., 2021). When treated with the CTLA4 negative/PD1 positive, CTLA4 positive/PD1 negative, and CTLA4 positive/PD1 positive immunity therapy regimens, the IPS of patients with high expression of ABI3BP was greater than that of those with low expression of ABI3BP. ABI3BP might provide a novel immunotherapeutic target for lung cancer. To understand the specific involvement of ABI3BP in tumor immunity micro-environments, however, additional study is required.

We now know more about the connection between ABI3BP and lung cancer thanks to this research, however, there are yet some limitations. Initially, the biological relevance of ABI3BP in LUAD and LUSC was examined in detail, but the expression and prognostic score of ABI3BP in small cell lung carcinoma were not examined. Through bio-informatics, ABI3BP has been connected with lung cancer. However, its molecular processes and involvement in tumor metastasis and the infiltration of the immune system remain unknown. Thirdly, ABI3BP was discovered to be related with immune infiltration in patients with lung carcinoma, however subgroup studies of immunity would have increased the data relevance. Overall, our findings indicate that ABI3BP is downregulated in lung tumors and linked with the diagnostic traits and prognosis of lung tumor patients. Expression of ABI3BP is intimately related to the immune invasion of lung tumor cells and may influence prognosis in part through modulating the immune invasion. ABI3BP might be employed as a biomarker of lung cancer prognosis related with immune infiltration. As a consequence, we can quantify expression of ABI3BP in surgical specimens of patients with lung tumor in order to identify the degree of malignancy, estimate a patient’s prognosis, and better evaluate the condition of the immune micro-environment, as well as create ABI3BP-targeted immunity therapy medications. We anticipate further research to unravel the biological activities of ABI3BP for the prognosis and the immunological micro-environment of lung malignancy patients.

## Data Availability

The original contributions presented in the study are included in the article/Supplementary Material, further inquiries can be directed to the corresponding author.

## Supplementary material

Supplementary Figure S1 Analysis of co-expression network of ABI3BP gene in LUSC. **(A)** Venn diagram of genes intersection of the Extracellular matrix protein coding genes and ABI3BP positively correlated genes. **(B)** Venn diagram of genes intersection of the Extracellular matrix protein coding genes and ABI3BP negatively correlated genes. The top 20 genes positively **(C)** and negatively **(D)** related with ABI3BP. **(E)** Identification of the hub genes. **(F)** GO & KEGG enrichment analysis of the hub genes in LUSC.

Supplementary Figure S2 The protein network of the ABI3BP-related genes and the mutation of the hub genes. **(A)** A network of 544 proteins in LUAD that are functionally similar to ABI3BP. **(B)** LUSC protein network of 563 functionally similar genes to ABI3BP. **(C)** SNV mutation frequency of hub genes in LUSC. **(D)** Oncoplot provides the situation of the SNV of the hub genes.

Supplementary Figure S3 Relationship between ABI3BP and immunoinfiltration in LUSC. **(A)** TME score of ABI3BP in LUSC. CIBERSORT **(B)**, XCELL **(C)**, QUANTISEQ **(D)**, MCPCOUNTER **(E)**, and EPIC **(F)** analysis between ABI3BP and tumor immune cells in LUSC.

Supplementary Figure S4 Relation of ABI3BP level of expression and lymphocyte, immunity modulator and chemokine in LUSC on the basis of TISIDB repository. **(A)** Relation of the abundance of tumor-infiltrating lymphocytes (TIL) and ABI3BP (6 TIL with the greatest correlation); **(B–D)** Relation of immunomodulators and ABI3BP (6 immunomodulators with the greatest correlation); **(E,F)** Relation of chemokines (or receptors) and ABI3BP (6 chemokines (or receptors) with the greatest correlation).
